# Impact of Perinatal Dioxin Exposure on Infant Growth: A Cross-Sectional and Longitudinal Studies in Dioxin-Contaminated Areas in Vietnam

**DOI:** 10.1371/journal.pone.0040273

**Published:** 2012-07-16

**Authors:** Muneko Nishijo, Pham The Tai, Hideaki Nakagawa, Shoko Maruzeni, Nguyen Thi Nguyet Anh, Hoang Van Luong, Tran Hai Anh, Ryumon Honda, Yuko Morikawa, Teruhiko Kido, Hisao Nishijo

**Affiliations:** 1 Department of Epidemiology and Public Health, Kanazawa Medical University, Uchinada, Ishikawa, Japan; 2 Biomedical and Pharmaceutical Research Center, Vietnam Military Medical University, Ha Noi, Vietnam; 3 School of Nursing, Kanazawa Medical University, Uchinada, Ishikawa, Japan; 4 Faculty of Health Sciences, Institute of Medical, Pharmaceutical and Health Sciences, Kanazawa University, Kanazawa, Ishikawa, Japan; 5 System Emotional Science, Graduate School of Medicine, University of Toyama, Toyama, Japan; Indiana University, United States of America

## Abstract

Dioxin exposure levels remain elevated in residents living around former US Air Force bases in Vietnam, indicating potential adverse impacts on infant growth. In this study, 210 mother–infant pairs in dioxin-contaminated areas in Vietnam were recruited at the infants’ birth and followed up for 4 months. Perinatal dioxin exposure levels were estimated by measurement of polychlorinated dibenzo-p-dioxins/furans toxic equivalent (PCDDs/Fs-TEQ) in breast milk. The infants’ size was measured at birth and 1 and 4 months after birth, and neurodevelopment was evaluated using the Bayley Scales III at 4 months of age. Among 4 dioxin groups (<25, 25–50, 50–75, ≥75 percentile of PCDDs/Fs-TEQ), cross-sectional comparisons of body size and neurodevelopment scales and comparisons of longitudinally assessed body size were performed respectively. At birth, head circumference of girls in the ≥75 percentile group was significantly larger than those in the <25 and 50–75 percentile groups. At 4 months of age, the weight and body mass index (BMI) of boys in the ≥75 percentile group were significantly lower than those in the other groups. Increase in weight was significantly lower in the ≥75 percentile group in both sexes from birth to 1 month but only in boys at 1–4 months of age. Estimated marginal mean values in a mixed model of weight and BMI during the first 4 months of life were significantly lower in the ≥75 percentile group in boys. In girls, marginal mean values for head circumference were increased with increase in dioxin levels. Only in boys, cognitive, language, and fine motor scores in the ≥75 percentile group were significantly lower than those in the other groups. These results suggested a considerable impact of perinatal dioxin exposure on infant growth, particularly in boys exposed to dioxins at high level of PCDDs/Fs-TEQ.

## Introduction

During the Vietnam War from 1961 to 1972, herbicides were sprayed in copious amounts in Southern Vietnam, contaminating the area with 2,3,7,8-tetrachlorodibenzo-*p*-dioxin (2,3,7,8-TetraCDD), the most toxic dioxin congener. After several decades of herbicide spraying, the concentrations of dioxins in the environment and in humans residing the sprayed areas remained elevated compared to those in the unsprayed areas [1]–[3]. Recently, the military airbases formerly used for storing herbicides were characterized as “hot spots” of dioxin contamination because of extremely high levels of dioxins in the soil samples [4]. The residents around these hot spots are thus currently exposed to high levels of dioxins. The dioxin levels in breast milk of lactating mothers in the hot spots were reported to be approximately 4-fold higher than those of lactating mothers residing in unsprayed areas [5]. Thus, Vietnamese infants are also exposed to dioxin during pregnancy via the placenta and postnatally through breastfeeding.

Infant body size at birth has been suggested as a good marker of fetal development. Growth in the neonatal period is also affected by fetal condition. Previously, in the Netherlands and Japan, small body size at birth and slightly delayed neurodevelopment in early life have been reported in infants exposed to dioxins and polychlorinated biphenyls (PCBs) at background levels during pregnancy [6]–[10]. Growth rate during infancy has also been suggested to be affected by dioxins and PCB exposure during the pre and postnatal periods [6]. However, effects of dioxins on infant growth, including body size after birth and neurodevelopment, have not been investigated in Vietnamese infants exposed to dioxins in the environment. The dose-effect relationships between infant growth and dioxin exposure levels have not been previously investigated.

Therefore, in this study, we investigated relationships between perinatal dioxin exposure levels, estimated from dioxin levels in breast milk, and infant growth and neurodevelopment. Subjects were infants whose mothers were residing in the “hot spots” of dioxin contamination in Southern Vietnam.

## Methods

### Study Location

The Thanh Khe and Son Tra districts in Da Nang City, located within 10 km of Da Nang airbase, which have been characterized as dioxin-contaminated areas, were selected for study area. In collaboration with Vietnamese government, Hatfield Consultants monitored the areas around the former Da Nang airbase in 2006 and 2009, and reported significant quantities of 2,3,7,8-TetraCDD in the soil samples (858–361,000 pg/g dry weight) from the northern end of Da Nang airport where mixing and loading areas and storage of herbicides were once located. This substance was also found in the sediment samples (674–8,580 pg/g dry weight) from Sen Lake and the drainage system from the north of the airbase to Sen Lake [11]. In addition, contamination due to other hazardous substances that contribute to the overall load of polychlorinated dibenzo-p-dioxins and dibenzofurans (PCDDs) and polychlorinated dibenzofurans (PCDFs), including PCBs, organochlorine pesticides, and hydrocarbons, was also observed [11].

### Study Population

One hundred and fifty-nine pregnant mothers who gave birth in the Thanh Khe district hospital (TK hospital) from July 2008 to January 2009 and 80 mothers who gave birth in the Son Tra district hospital (ST hospital) from November to December 2009 were recruited by obstetricians on the bases of the following criteria: the mothers residing in the Thanh Khe and Son Tra districts during pregnancy, they gave birth to full-term babies and the delivery was free of any complications. When the infants were 1 month (30 days) old, nurses from communal health centers visited them and collected breast milk samples from 147 mothers (93%) in Thanh Khe and 80 mothers (100%) in Son Tra. Infant size including weight, length (height), and head and abdominal circumferences were measured by the nurses and doctors at birth and 1 and 4 months after birth. When the infants were 4 months old, their neurodevelopment was evaluated using the Bayley Scales of Infant and Toddler Development, third edition (Bayley III; Pearson Education, Inc., San Antonio, TX, USA). Body mass index (BMI) was obtained using the formula: weight/(length)^2^, and head circumference/length (HL ratio) was calculated to evaluate infant proportions. A total of 219 mother–infant pairs participated in the survey. However, the data of nine mothers lacked information about body build and socioeconomic factors. Therefore, the data for 210 mother-infant pairs were used for analysis in the present study.

Information about the mothers regarding age, history of residency, parity, smoking and drinking habits, job, education, family income and smoking habits, and duration of breastfeeding was collected through interviews. Height and weight of the mothers, information about the infants (number of gestational weeks at birth and gender), and details of complications during pregnancy and delivery were collected from the obstetricians. Seventy percent of the 210 subjects were housewives, and the others were saleswoman, factory workers, and office workers with no occupational history of pesticide exposure. All infants were breastfed until 4 months of age. No significant differences in gestational age and birth weight were observed between the infants who participated in the present follow-up study and those who did not.

Written informed consent was obtained from all the mothers. The institutional ethics board of epidemiological studies at Kanazawa Medical University approved the study design (License number: ES-74). The Department of Health and Prevention of Diseases, Da Nang City government in Vietnam reviewed and approved the informed consent process.

### Breast Milk Collection and Quantification of PCDDs/Fs in Breast Milk

Breast milk samples from nursing mothers were collected in clean polyethylene containers when the infants were 1 month old. Samples were collected at home by hand pressure with the assistance of midwives or medical workers. Each sample (approximately 20 mL) was frozen, transported in dry ice to Japan, and stored at −30°C until analysis.

Approximately 10 mL of breast milk from each sample was used to quantify the levels of seven congeners of PCDDs and 10 congeners of PCDFs; 2,3,7,8-TetraCDD, 1,2,3,7,8-PentaCDD, 1,2,3,4,7,8-HexaCDD, 1,2,3,6,7,8-HexaCDD, 1,2,3,7,8,9-HexaCDD, 1,2,3,4,6,7,8-HeptaCDD, OctaCDD, 2,3,7,8-TetraCDF, 1,2,3,7,8-PentaCDF, 2,3,4,7,8-PentaCDF, 1,2,3,4,7,8-HexaCDF, 1,2,3,6,7,8-HexaCDF, 1,2,3,7,8,9-HexaCDF, 2,3,4,6,7,8-HexaCDF, 1,2,3,4,6,7,8-HeptaCDF, 1,2,3,4,7,8,9-HeptaCDF and OctaCDF. Lipid content in breast milk was extracted and treated in a series of purifying operations, including alkali digestion and hexane extraction. Chromatography was performed on a multi-layered silica gel column. A single-layered column of activated carbon dispersed on silica gel was used to separate and collect PCDDs/Fs fractions. Quantification was performed using a gas chromatograph (HP-6980; Hewlett-Packard, Palo Alto, CA, USA) equipped with a high-resolution mass spectrometer (MStation-JMS700, JEOL, Tokyo, Japan) operating in the selected ion monitoring mode. The gas chromatograph was equipped with an ENV-5MS column (Kanto Chemical Co., Inc., Tokyo, Japan). All samples were analyzed at the Center for High Technology, Kanazawa Medical University, Japan. The established method of analysis has been described previously [12].

Lipid-based concentrations of the 17 congeners of PCDDs/Fs were calculated. Toxic equivalent factors (TEFs) for each congener were referenced from the list of WHO 2005 TEF [13]. Toxic equivalents (TEQ) of PCDDs and PCDFs (PCDDs-TEQ and PCDFs-TEQ) were calculated by multiplying each congener concentration with its TEF and summing the values. The concentrations of congeners below detection limits were set at half the detection limits.

### Bayley Scales of Infant and Toddler Development (Bayley III)

The Bayley III is an individually administered scale for assessment of developmental functioning of infants and young children aged 1–42 months across the cognitive, language, and motor domains. The language and motor domains have two subscales: receptive and expressive communication (language) and fine and gross motor skills. In administering the scales, the examiner observed or used various test materials to interact with the infants, and scored them based on their responses. When the infants were 4 months old, they were tested by one examiner in the presence of the infants’ caregivers. Before starting the survey, the examiner was well trained for evaluation using the scales by a senior examiner. The unique rules of administration were strictly followed, as described in the guidance manual of the Bayley III. A trial survey of a group of 18 Vietnamese infants was performed to assure the feasibility of the Bayley III in the Vietnamese population. Evidence of convergent and discriminant validity of the test was confirmed by checking the intercorrelations among subtests, based on a priori hypothesis that subtests designed to measure similar underlying constructs would have higher correlations than subtests that were designed to measure dissimilar constructs. The results of Bayley III testing in the present study were in agreement with the hypothesis (Bayley III technical manual, Chapter 5: Evidence of Validity).

### Data Analysis

The SPSS (ver. 11.0) software package for Windows (SPSS; Chicago, IL, USA) was used for statistical analyses. Subjects were divided into four groups according to PCDDs/Fs-TEQ levels in breast milk (<25, 25–50, 50–75 and ≥75 percentile of 210 samples). Means of characteristic factors of mother-infant pairs and dioxin levels in breast milk were compared among the four PCDDs/Fs-TEQ groups using one-way ANOVA, and followed by Scheffé’s test for comparisons between groups. Cross-sectional comparisons of infant body size and scores on the Bayley III among the 4 PCDDs/Fs-TEQ groups at birth and 1 and 4 months after birth were performed using a generalized linear model after adjustment for confounding factors in each gender. To evaluate relationships between PCDDs/Fs-TEQ levels in breast milk and longitudinally assessed growth measurements, multivariate mixed-effects regression models for repeated measures were performed for weight, length, BMI, and head circumference after conversion of z scores consistent with the WHO infant growth standard (www.who.int/childgrowth/standards). Before performing multivariable analysis, confounding factors were identified for covariates based on identification of a significant correlation coefficient with at least one aspect of infant development (P<0.05, Pearson’s correlation) and with reference to factors with meaningful influence on infant growth and neurodevelopment established in previous reports [14], [15].

## Results

Table 1 lists the characteristics of the 210 mother–infant pairs, including mean values for residency, maternal age, parity, maternal educational years, family income, alcohol drinking during pregnancy, family smoking, maternal body size, gestational weeks, infant gender, and incidence of low birth weight (<2500 g). Mean values for residency in the 25–50, 50–75 and ≥75 percentile groups were significantly longer than that in the <25 percentile group. Mother’s age in the ≥75 percentile group was significantly higher than that in the 25–50 and <25 percentile groups. Primipara mothers made up 29.5% of all mother–infant pairs. During pregnancy, 17.1% of the mothers consumed alcohol, but only occasionally (< once a week, 1–2 glasses of beer at a time). None of the mothers were smokers, but 81.9% of the mothers lived with family members who smoked. Family income was measured by earnings of both parents in Vietnam Dong in 1 month. No significant difference was observed in alcohol consumption, family smoking rates, and family income among the four groups.

**Table 1:** Characteristics of mothers and infants in groups with different PCDDs/Fs-TEQ levels.

Percentile of PCDDs/Fs-TEQ (pg/gfat)			<25		25–50			50–75			≥75			Total			
Number of subjects			52		53			52			53			210			
Subject	Characteristicfactor	Unit	Mean	SD	Mean	SD	p	Mean	SD	p	Mean	SD	p	Mean	SD	Min.	Max.
Mothers	Residency	years	12.7	10.6	19.7	10.8	a	21.0	12.5	a	23.6	10.4	a	19.2	11.7	1	43
	Age	years	26.1	4.6	26.4	5.5		28.6	6.5		30.8	6.0	a,b	28.0	6.0	16	43
	Parity(rate of primipara)	%	21.2		32.1			36.5			28.3			29.5			
	Education	years	8.6	3.1	8.8	3.8		8.8	3.4		8.2	3.7		8.6	3.5	0	17
	Income	x1000D/mon	3049	1990	2918	1372		2997	1473		2887	1577		2962	1608	500	10000
	Alcohol drinking(yes/no)	%	23.1		20.8			17.3			7.5			17.1			
	Family smoking(yes/no)	%	80.8		84.9			84.6			77.4			81.9			
	Weight before delivery	kg	56.6	7.2	58.7	7.2		58.9	6.4		56.7	6.6		57.7	6.9	41	75
	Height	cm	152.9	4.5	154.9	5.6		154.6	4.9		153.6	5.0		154.0	5.0	141	176
Infants	Gestational period	weeks	39.5	0.8	39.7	0.8		39.4	0.7		39.6	0.9		39.5	0.8	37	42
	Gender(rate of boys)	%	57.7		69.8			53.8			47.2			57.1			
	Low birth weight<2500g	%	0.0		0.0			0.0			1.9			0.5			
Breast milk*	2,3,7,8-TetraCDD	pg/gfat	0.70	2.01	1.24	1.81	a	1.82	1.70	a,b	2.83	1.82	a,b,c	1.46	2.21	0.13	9.98
	TEQ%	%	12.2	7.8	12.4	5.9		13.4	6.3		14.1	7.1		13.1	6.8	1.1	53.7
	PCDDs-TEQ	pg-TEQ/gfat	3.80	1.35	6.63	1.16	a	8.58	1.15	a,b	12.92	1.34	a,b,c	7.28	1.65	1.90	39.39
	PCDFs-TEQ	pg-TEQ/gfat	3.66	1.37	5.95	1.17	a	8.25	1.15	a,b	11.93	1.28	a,b,c	6.82	1.63	1.81	32.94
	PCDDs/Fs-TEQ	pg-TEQ/gfat	7.53	1.31	12.66	1.10	a	16.93	1.09	a,b	25.09	1.26	a,b,c	14.22	1.61	3.73	72.34

a: p<0.01 as compared with <25percentile group, b: p<0.01 as compared with 25-50 percentile group, c: p<0.01 as compared with 50-75 percentile group (tested by Scheffe test).

SD: standard deviation, p: p-value, Min.: minimum, Max.: maximum, x1000D/mon: 1000 Vietnamese Dong per month, TEQ: toxic equivalent.

* : geometrical mean and geometrical standard deviation.

Concentrations and TEQ% of 2,3,7,8-TetraCDD, PCDDs-TEQ and PCDFs-TEQ among the four PCDDs/Fs-TEQ groups were compared (Table 1). Levels of PCDDs-TEQ, PCDFs-TEQ and concentrations of 2,3,7,8-TetraCDD significantly increased with increasing levels of PCDDs/Fs-TEQ, but no significant difference in TEQ% of 2,3,7,8-TetraCDD was found among the four groups.

To assess the effects of dioxin exposure on infant build, infant weight at birth and infant weight 1 and 4 months after birth were compared among the four PCDDs/Fs-TEQ groups after adjusting for maternal age, weight, parity (primipara or not), educational period (years), alcohol drinking during pregnancy (yes/no), family income, family smoking (yes/no), gestational weeks at birth, infant age (days) on the day of each examination, and location difference (TK or ST hospital) in boys (Table 2) and in girls (Table 3). In this model, maternal weight was selected as a confounding factor because its correlation with infant build was higher than that of maternal height. Although no mother smoked, family smoking was included because family smoking is also related to lower birth weight. No significant difference in absolute values and age-adjusted z scores for all body size parameters at birth was observed among the four PCDDs/Fs-TEQ groups in boys. Moreover, at 1 month of age, no significant difference in body size among the four dioxin groups was observed in boys except a difference in HL ratio between the 25–50 and 50–75 percentile groups. At 4 months of age, only in boys, absolute values and age-adjusted z scores for weight and BMI and abdominal circumference in the ≥75 percentile group were lower than those in the other dioxin groups, with significant differences as compared with those in the 25–50 and 50–75 percentile groups. In addition, HL ratio in the ≥75 percentile group was higher than that in the 50–75 percentile group. HL ratio in the ≥75 percentile group was the lowest among the four dioxin groups, but no significant difference was found as compared with the other groups. No clear relationship was observed between length and head circumference and dioxin levels in boys at 4 month of age (Table 2). On the other hand, in girls (Table 3), absolute values and age-adjusted z scores for weight and BMI and abdominal circumference at birth in the ≥75 percentile group were significantly higher than those in the 50–75 percentile group, but no difference was found compared with the other groups. However, in girls, absolute value and age adjusted z score for head circumference and HL ratio at birth were higher with high dioxin levels. These parameters in the ≥75 percentile groups were significantly higher than those in the <25 and 50–75 percentile groups, suggesting that newborns with high dioxin exposure had larger head proportions. At 1 month of age, absolute values and age-adjusted z scores for weight in the 50–75 percentile group were significantly lower than those in the <25 and 25–50 percentile groups, but no significant difference was observed between the ≥75 percentile group and the other groups. Moreover, at 4 months of age, no significant difference in body size among the four dioxin groups was observed in girls after adjusting for confounding factors (Table 3).

**Table 2:** Comparisons of body size in boys after adjusting for confounders among 4 dioxin groups.

Percentile of PCDDs/Fs-TEQ (pg/gfat)			<25		25–50			50–75			≥75		
Number of subjects			30		37			28			25		
Infant age	Parameter	Unit	Mean	SE	Mean	SE	p	Mean	SE	p	Mean	SE	p
At birth	Weight	abs. value (g)	3289.9	73.0	3250.0	63.6		3253.3	73.0		3278.7	83.6	
		age adj. Z	−0.226	0.154	−0.341	0.134		−0.309	0.154		−0.281	0.177	
	Length	abs. value (cm)	50.1	0.32	50.1	0.28		49.5	0.32		49.7	0.37	
		age adj. Z	0.060	0.171	0.048	0.149		−0.225	0.171		−0.122	0.196	
	Head	abs. value (cm)	33.8	0.30	33.7	0.26		33.0	0.30		33.5	0.34	
		age adj. Z	−0.394	0.235	−0.516	0.205		−1.024	0.235		−0.786	0.269	
	BMI	abs. value	13.1	0.25	12.9	0.22		13.2	0.25		13.2	0.29	
		age adj. Z	−0.296	0.195	−0.437	0.170		−0.178	0.195		−0.214	0.224	
	Abdominal	abs. value (cm)	32.3	0.37	32.0	0.32		31.8	0.37		32.2	0.42	
	Head/Length	abs. value	0.675	0.006	0.673	0.005		0.667	0.006		0.673	0.006	
1 month	Weight	abs. value (g)	4542.5	104.8	4498.3	90.5		4501.4	102.5		4328.0	117.9	
		age adj. Z	0.292	0.175	0.219	0.151		0.224	0.171		−0.065	0.197	
	Length	abs. value (cm)	56.1	0.42	55.5	0.36		56.1	0.41		55.7	0.47	
		age adj. Z	1.204	0.219	0.799	0.189		1.108	0.214		0.928	0.247	
	Head	abs. value (cm)	37.4	0.25	37.4	0.22		36.9	0.25		37.0	0.28	
		age adj. Z	0.454	0.206	0.454	0.178		0.065	0.202		0.181	0.232	
	BMI	abs. value	14.3	0.28	14.6	0.24		14.3	0.28		13.9	0.32	
		age adj. Z	−0.430	0.223	−0.247	0.193		−0.448	0.218		−0.792	0.251	
	Abdominal	abs. value (cm)	37.6	0.50	38.2	0.43		38.0	0.49		37.5	0.56	
	Head/Length	abs. value	0.666	0.005	0.674	0.004		0.659	0.005	c	0.665	0.006	
4 months	Weight	abs. value (g)	6927.0	142.9	6990.0	124.8		7222.9	143.6		6562.1	163.6	c,f
		age adj. Z	−0.191	0.183	−0.191	0.160		0.179	0.183		−0.676	0.214	f
	Length	abs. value (cm)	64.7	0.37	64.0	0.32		65.3	0.37		64.3	0.42	
		age adj. Z	0.260	0.182	−0.190	0.159		0.485	0.182	d	−0.037	0.212	
	Head	abs. value (cm)	41.8	0.22	41.7	0.20		41.4	0.22		41.8	0.26	
		age adj. Z	−0.102	0.199	−0.343	0.174		−0.401	0.200		−0.125	0.233	
	BMI	abs. value	16.5	0.30	17.1	0.26		16.9	0.30		15.9	0.34	c,e
		age adj. Z	−0.510	0.211	−0.152	0.184		−0.163	0.211		−0.931	0.246	c,e
	Abdominal	abs. value (cm)	41.7	0.48	42.6	0.42		43.3	0.48	a	40.8	0.55	c,f
	Head/Length	abs. value	0.646	0.004	0.652	0.004		0.634	0.004	d	0.651	0.005	e

a: p<0.05, b: p<0.01 as compared with <25 percentile group, c: p<0.05, d: p<0.01 as compared with 25–50 percentile group, e: p<0.05, f: p<0.01 as compared with 50–75 percentile group.

Mean: adjusted mean, SE: standard error, p: p-value, abs. value: absolute value, age adj. Z: age adjusted z score.

Confounders for measurements of body size: parity (primipara or not), maternal age, weight, educational period (years), alcohol drinking during pregnancy (yes/no), family income, family smoking (yes/no), gestational weeks, infant age (days) on the day of each examination, and location difference (TK or ST hospital).

Confounders for z scores of body size: parity (primipara or not), maternal age, weight, educational period (years), alcohol drinking during pregnancy (yes/no), family income, family smoking (yes/no), gestational weeks, and location difference (TK or ST hospital).

Note: Body size at 1 month was missing for 2 boys.

**Table 3:** Comparisons of body size in girls after adjusting for confounders among 4 dioxin groups.

Percentile of PCDDs/Fs-TEQ (pg/gfat)			<25		25–50			50–75			≥75		
Number of subjects			22		16			24			28		
Infant age	Parameter	Unit	Mean	SE	Mean	SE	p	Mean	SE	p	Mean	SE	p
At birth	Weight	abs. value (g)	3176.1	65.6	3257.1	70.9		3025.1	59.4	c	3270.6	55.6	f
		age adj. Z	−0.204	0.149	−0.024	0.161		−0.535	0.135	c	0.020	0.126	f
	Length	abs. value (cm)	49.5	0.33	49.4	0.35		49.0	0.30		49.7	0.28	
		age adj. Z	0.281	0.174	0.245	0.188		0.051	0.157		0.435	0.160	
	Head	abs. value (cm)	32.6	0.29	33.2	0.32		33.1	0.27		33.9	0.25	b,e
		age adj. Z	−1.106	0.247	−0.638	0.267		−0.641	0.224		−0.032	0.209	b,e
	BMI	abs. value	12.9	0.24	13.3	0.26		12.6	0.22	c	13.2	0.20	e
		age adj. Z	−0.384	0.205	−0.084	0.221		−0.690	0.185	c	−0.172	0.173	e
	Abdominal	abs. value (cm)	31.7	0.36	31.7	0.39		31.1	0.32		32.6	0.30	f
	Head/Length	abs. value	0.658	0.006	0.671	0.007		0.676	0.006		0.681	0.005	a
1 month	Weight	abs. value (g)	4217.0	79.5	4255.7	85.7		3979.8	71.6	a,c	4125.5	68.7	
		age adj. Z	0.162	0.146	0.226	0.157		−0.275	0.131	a,c	−0.008	0.126	
	Length	abs. value (cm)	55.0	0.55	55.8	0.59		54.6	0.50		55.1	0.48	
		age adj. Z	1.077	0.286	1.498	0.308		0.857	0.258		1.157	0.247	
	Head	abs. value (cm)	36.2	0.30	36.9	0.32		36.3	0.27		36.5	0.26	
		age adj. Z	0.345	0.249	0.886	0.269		0.460	0.225		0.606	0.215	
	BMI	abs. value	13.9	0.29	13.7	0.31		13.4	0.26		13.5	0.25	
		age adj. Z	−0.381	0.220	−0.555	0.237		−0.796	0.198		−0.666	0.190	
	Abdominal	abs. value (cm)	36.7	0.48	37.3	0.51		36.7	0.43		37.1	0.41	
	Head/Length	abs. value	0.660	0.007	0.662	0.007		0.667	0.006		0.663	0.006	
4 months	Weight	abs. value (g)	6310.6	146.7	6362.1	151.3		6215.3	128.3		6338.7	120.5	
		age adj. Z	−0.292	0.191	−0.184	0.206		−0.338	0.173		−0.212	0.162	
	Length	abs. value (cm)	63.2	0.43	63.6	0.45		62.7	0.38		62.6	0.36	
		age adj. Z	0.491	0.211	0.646	0.228		0.203	0.191		0.171	0.179	
	Head	abs. value (cm)	40.6	0.29	40.6	0.29		40.5	0.25		40.5	0.23	
		age adj. Z	−0.326	0.237	−0.253	0.255		−0.371	0.214		−0.362	0.200	
	BMI	abs. value	15.8	0.31	15.7	0.32		15.8	0.27		16.1	0.25	
		age adj. Z	−0.682	0.199	−0.683	0.215		−0.570	0.180		−0.336	0.168	
	Abdominal	abs. value (cm)	40.8	0.57	40.5	0.59		40.9	0.50		41.0	0.47	
	Head/Length	abs. value	0.643	0.005	0.639	0.005		0.646	0.004		0.647	0.004	

a: p<0.05, b: p<0.01 as compared with <25 percentile group, c: p<0.05, d: p<0.01 as compared with 25–50 percentile group, e: p<0.05, f: p<0.01 as compared with 50–75 percentile group.

Mean: adjusted mean, SE: standard error, p: p-value, abs. value: absolute value, age adj. Z: age adjusted z score.

Confounders for measurements of body size: parity (primipara or not), maternal age, weight, educational period (years), alcohol drinking during pregnancy (yes/no), family income, family smoking (yes/no), gestational weeks, infant age (days) on the day of each examination, and location difference (TK or ST hospital).

Confounders for z scores of body size: parity (primipara or not), maternal age, weight, educational period (years), alcohol drinking during pregnancy (yes/no), family income, family smoking (yes/no), gestational weeks, and location difference (TK or ST hospital).

Note: Body size at 1 month was missing for 1 girl.

Because growth velocity of body size measurements is a good marker for infant growth, we compared estimated increases in weight, height, and head and abdominal circumference per day during the first month after birth and at 1–4 months among the 4 PCDDs/Fs-TEQ groups after adjusting for the same confounding factors that were included in the body size analysis (Table 4). In boys, the weight gain (g/day) from birth to 1 month in the ≥75 percentile group was lower than that in the <25 and 25–50 percentile groups. In girls, the weight gain from birth to 1 month in the ≥75 percentile group was lower than that in the <25 percentile group. During the period from 1 to 4 months, increases in weight (g/day) and abdominal circumference (cm/day) in the ≥75 percentile group were significantly lower than those in the <25 or 50–75 percentile groups in boys, whereas growth rates were similar in the 50–75, 25–50 and <25 percentile groups. However, no significant difference in growth velocity during the period from 1 to 4 months among the four PCDDs/Fs-TEQ groups was found in girls.

**Table 4:** Comparisons of estimated body growth per day after adjusting for confounders among 4 dioxin groups.

Percentile of PCDDs/Fs-TEQ (pg/gfat)				<25		25–50			50–75			≥75		
Observation period	Gender	Parameter	Unit	Mean	SE	Mean	SE	p	Mean	SE	p	Mean	SE	p
Birth to 1 month	Boys	N		29		36			28			25		
		Weight	g/day	42.4	2.4	42.1	2.1		41.6	2.3		34.7	2.7	a,b
		Length	cm/day	0.20	0.01	0.18	0.01		0.22	0.01	b	0.20	0.02	
		Head	cm/day	0.12	0.01	0.12	0.01		0.13	0.01		0.12	0.01	
		Abdominal	cm/day	0.17	0.02	0.21	0.01		0.21	0.02		0.18	0.02	
	Girls	N		22		16			24			27		
		Weight	g/day	34.9	2.3	33.5	2.5		31.9	2.1		28.5	2.0	a
		Length	cm/day	0.19	0.02	0.21	0.02		0.18	0.02		0.18	0.02	
		Head	cm/day	0.12	0.01	0.12	0.01		0.11	0.01		0.09	0.01	
		Abdominal	cm/day	0.17	0.02	0.18	0.02		0.19	0.02		0.15	0.02	
1 to 4 months	Boys	N		29		36			28			25		
		Weight	g/day	28.4	2.0	28.1	1.7		29.4	2.0		22.8	2.2	c
		Length	cm/day	0.13	0.02	0.11	0.02		0.09	0.02		0.08	0.02	
		Head	cm/day	0.07	0.01	0.06	0.01		0.05	0.01		0.04	0.01	
		Abdominal	cm/day	0.07	0.01	0.06	0.01		0.06	0.01		0.03	0.02	a
	Girls	N		22		16			24			27		
		Weight	g/day	21.7	1.9	22.2	2.0		25.5	1.7		26.2	1.6	
		Length	cm/day	0.08	0.02	0.08	0.02		0.10	0.02		0.11	0.02	
		Head	cm/day	0.04	0.01	0.04	0.01		0.05	0.01		0.06	0.01	
		Abdominal	cm/day	0.04	0.01	0.04	0.01		0.05	0.01		0.06	0.01	

a: p<0.05 as compared with <25 percebtile group, b: p<0.05 as compared with 25–50 percentile group, c: p<0.05 as compared with 50–75 percentile group.

N: number of subjects, Mean: adjusted mean, SE: standard error, p: p-value.

Confounders: parity (primipara or not), maternal age, weight, educational period (years), alcohol drinking during pregnancy (yes/no), family income, family smoking (yes/no), gestational weeks, infant age (days) on the day of each examination, and location difference (TK or ST hospital).

Note: Body size at 1 month was missing for 2 boys and 1 girl.

Relationships between PCDDs/Fs-TEQ levels and adjusted z scores for weight, height, BMI, and head circumference from birth to 4 months were analyzed using a linear mixed model for repeated measures after adjusting for maternal age, weight, parity, educational period, drinking habit, family income, family smoking, gestational weeks at birth, and location difference. ANOVA test results for fixed factors (PCDDs/Fs-TEQ categories) and estimated marginal means for each PCDDs/Fs-TEQ category adjusted for confounding factors are shown in Table 5. Estimated adjusted mean values of weight and BMI in the ≥75 percentile group were significantly lower than those in the <25 percentile group, and significant associations of PCDDs/Fs-TEQ categories with infant weight and BMI were observed in boys. In contrast, among girls, adjusted marginal mean values of weight and BMI in the ≥75 percentile group were significantly higher than those in the <25 percentile group, although test results for associations of PCDDs/Fs-TEQ categories with BMI were not significant (ANOVA). However, PCDDs/Fs-TEQ categories were significantly associated with head circumference. Adjusted marginal mean values for head circumference were at higher levels of PCDDs/Fs-TEQ in girls, suggesting a dose-dependent response relationship between PCDDs/Fs-TEQ and head circumference.

**Table 5:** Association of dioxin levels with estimated marginal means of infant growth parameters.

		Boys				Girls			
Parameter	PCDDs/Fs-TEQ	N	Est. Mean	(95% CI)	p-value	N	Est. Mean	(95% CI)	p-value
Weight (z-score)	<25 percentile	30	−0.047	(−0.241, 0.148)	–	22	−0.280	(−0.474, −0.087)	–
	25–50	37	−0.050	(−0.225, 0.124)	0.978	16	0.053	(−0.174, 0.279)	0.028
	50–75	28	−0.021	(−0.221, 0.179)	0.858	24	−0.294	(−0.479, −0.109)	0.921
	≥75	25	−0.421	(−0.634, −0.208)	0.011	28	−0.010	(−0.182, 0.163)	0.040
	ANOVA				0.021				0.022
Length (z-score)	<25 percentile	30	0.317	(0.095, 0.538)	–	22	0.356	(0.111, 0.601)	–
	25–50	37	0.137	(−0.062, 0.336)	0.237	16	0.642	(0.354, 0.929)	0.137
	50–75	28	0.349	(0.120, 0.577)	0.842	24	0.289	(0.054, 0.523)	0.695
	≥75	25	0.133	(−0.111, 0.377)	0.274	28	0.470	(0.252, 0.688)	0.494
	ANOVA				0.380				0.265
BMI (z-score)	<25 percentile	30	−0.347	(−0.574, −0.120)	−	22	−0.661	(−0.888, −0.434)	–
	25–50	37	−0.207	(−0.411, −0.003)	0.366	16	−0.449	(−0.715, −0.183)	0.234
	50–75	28	−0.300	(−0.534, −0.067)	0.777	24	−0.586	(−0.803, −0.369)	0.639
	≥75	25	−0.756	(−1.005, −0.507)	0.017	28	−0.327	(−0.529, −0.124)	0.031
	ANOVA				0.008				0.139
Head (z-score)	<25 percentile	30	−0.034	(−0.286, 0.218)	–	22	−0.492	(−0.778, −0.207)	–
	25–50	37	−0.094	(−0.320, 0.133)	0.727	16	−0.019	(−0.353, 0.316)	0.035
	50–75	28	−0.409	(−0.668, −0.150)	0.042	24	−0.188	(−0.461, 0.085)	0.131
	≥75	25	−0.205	(−0.482, 0.072)	0.368	28	0.026	(−0.228, 0.280)	0.008
	ANOVA				0.180				0.047

Mixed effects repeated measures regression model adjusted for parity (primipara or not), maternal age, weight, educational period (years), alcohol drinking during pregnancy (yes/no), family income, family smoking (yes/no), gestational weeks, and location difference (TK or ST hospital).

N: number of subjects, Est. Mean: estimated adjusted marginal mean, CI: confidence interval.

P-value of each category is for comparison with <25 percentile group, and P-value of ANOVA is for type III test.

Differences in neurodevelopmental scores, as evaluated by the Bayley III, were investigated among the four PCDDs/Fs-TEQ groups at 4 months of age after adjustment for maternal age, weight, parity, educational period, drinking habit, family income, family smoking, gestational weeks at birth, infant age (days) at examination, and location difference. At this time, weight and BMI at 4 months of age were not included as confounding factors, because no significant relationship was evident between neurodevelopmental scores and anthropometric measurements. The results in both sexes are shown in Table 6. In boys, scores for cognitive, language, particularly expressive communication, and fine motor skills in the ≥75 percentile group were significantly lower than those in the 25–50 and 50–75 percentile groups. Only for expressive communication, scores in the ≥75 percentile group were significant lower than those in the <25 percentile group among boys. In girls, no significant difference was observed in neurodevelopmental scores for all domains except cognitive ability among the PCDDs/Fs-TEQ groups at 4 months of age. For cognitive ability, scores in the ≥75 percentile group were significantly lower than those in the 25–50 percentile group among girls.

**Table 6:** Comparisons of Bayley III scores after adjusting for confounders among 4 dioxin groups.

Percentile of PCDDs/Fs-TEQ (pg/gfat)				<25		25–50				50–75					≥75		
Gender	Domain			Mean	SE	Mean	SE	p		Mean		SE	p		Mean	SE	p
Boys	N			30		37				28					25		
	Cognitive	Total	cs	93.0	2.3	95.2	2.0		95.1		2.3			87.2		2.6	b,c
	Language	Total	cs	96.1	1.2	98.4	1.0		96.7		1.2			94.0		1.3	b
		Reseptive com	ss	8.4	0.3	9.1	0.2	a	8.5		0.3			8.4		0.3	
		Expressive com	ss	10.2	0.2	10.3	0.2		10.3		0.2			9.5		0.2	a,b,c
	Motor	Total	cs	101.1	2.0	102.4	1.7		101.1		2.0			96.9		2.2	
		Fine motor	ss	8.8	0.3	9.3	0.3		8.6		0.3			7.9		0.4	b
		Gross motor	ss	11.4	0.5	11.4	0.4		11.7		0.5			11.0		0.5	
Girls	N			22		15			24					28			
	Cognitive	Total	cs	95.0	3.0	100.2	3.3		93.8		2.6			91.1		2.5	b
	Language	Total	cs	98.3	1.7	97.0	1.8		95.7		1.4			95.3		1.4	
		Reseptive com	ss	8.9	0.4	8.5	0.4		8.4		0.3			8.4		0.3	
		Expressive com	ss	10.5	0.3	10.5	0.3		10.2		0.3			10.0		0.3	
	Motor	Total	cs	105.0	2.6	106.6	2.8		101.6		2.3			101.6		2.2	
		Fine motor	ss	9.9	0.5	10.2	0.5		9.2		0.4			9.0		0.4	
		Gross motor	ss	11.7	0.5	11.9	0.5		11.2		0.4			11.5		0.4	

a: p<0.05 as compared with <25 percentile group, b: p<0.05 as compared with 25–50 percentile group, c: p<0.05 as compared with 50–75 percentile group.

N: number of subjects, Mean: adjusted mean, SE: standard error, p: p-value.

cs: composite score, ss: scale score, com: communication.

Confounders: parity (primipara or not), maternal age, weight, educational period (years), alcohol drinking during pregnancy (yes/no), family income, family smoking (yes/no), gestational weeks, infant age (days) on the day of each examination, and location difference (TK or ST hospital).

Note: Bayley III test could not be performed for 1 girl.

## Discussion

### Dioxin Contamination of Breast Milk in Hot Spot Areas

Dioxin contamination in hot spot areas in southern Vietnam is originated from Agent Orange, a herbicide and defoliant sprayed by the U.S. during the Vietnam War. The high contribution of 2,3,7,8-TetraCDD to the total amount of dioxins (approximately 90%) was due to the fact that Agent Orange contained only 2,3,7,8-TetraCDD, and not other PCDD and PCDF congeners. However, in the subjects living in the hot spot areas of the present study, the most highest TEQ% of 2,3,7,8-TCDD was 14.1% in the 75 percentile group which was much less than that documented in previous studies [2], [16], [17]. In addition, no significant difference in TEQ% of 2,3,7,8-TetraCDD was found among the four PCDDs/Fs-TEQ groups. In a previous study, 520 breast milk samples from Vietnamese mothers living in hot spot areas, sprayed areas and unsprayed areas. TEQs of PCDDs/Fs in primiparae were 14.10, 10.89, and 4.09 pg/g of fat, with significant differences between the three areas, but 2,3,7,8-TetraCDD contributed only approximately 10% of total TEQ in both hot spot and sprayed areas [5]. The low contribution of 2,3,7,8-TetraCDD to PCDDs/Fs-TEQ in breast milk samples in the present study might be partly explained by chronological changes in congener composition when dioxins have passed through many organisms in the decades since spraying. Another explanation may be that the subjects are representative of the general population for whom direct contact with polluted soil and contaminated fresh water fish were prohibited. They eat seafood caught in the neighboring sea into which channel and river water from contaminated lakes flows, but which is also contaminated because of other sources. Sources of exposure in addition to Agent Orange and overall dioxin load remain to be determined in these subjects.

### Gender Differences in Dioxin Effects on Infant Growth

In the present study, maternal dioxin exposure affected infant weight and BMI in boys at 4 months after birth, because significantly low measurements were observed in boys exposed to higher levels of dioxins (PCDDs/Fs-TEQ ≥75 percentile). Incremental measurement of weight and abdominal circumference during the follow-up period resulted in significantly lower values among boys in the ≥75 percentile group, who were thinner at 4 months of age than the boys exposed to lower levels of dioxins. Not only somatic growth, but also neurodevelopment, particularly expressive language, at 4 months of age was relatively poor in boys exposed to higher levels of dioxins. In contrast, longitudinal measurements of weight and BMI for 4 months in girls exposed to higher levels (≥75 percentile) of dioxins were higher than for those exposed to lower levels. However, the increased values for weight and abdominal circumference during the neonatal period were significantly lower in girls exposed to higher levels of dioxins. Moreover, no difference was observed in neurodevelopment among female infants at different TEQ levels.

These results suggest that perinatal dioxin exposure, estimated using PCDDs/Fs-TEQ levels in maternal breast milk, significantly affected infant growth, including body size and neurodevelopment, in early life particularly in boys. Similar gender-specific differences (boys are more susceptible to growth restriction than girls) were reported in infants whose mothers were Yusho patients accidentally exposed to rice oil contaminated with PCDDs/Fs and PCB [18].

In girls, head circumference at birth significantly increased with increasing dioxin levels, showing a dose–effect relationship. HL ratio was higher, suggesting a larger proportion of head to body. Head circumference is an index for estimating brain weight in newborns [19], and infant head circumference at birth can be affected by environmental factors during pregnancy, such as heavy smoking [20] and nutritional status [21], [22]. Inverse relationships between 2,3,7,8-TCDD in maternal breast milk and infant head circumference at birth in a Japanese population have been previously reported [23]. However, the present result suggested unusual head growth in girls associated with dioxin TEQ levels, which was reversible in the first month after birth and had no effect on neurodevelopment. Longer follow up is necessary to clarify the outcome of this evidence in girls, because increased head circumference at birth has been reported to be a risk factor for brain cancer during childhood [24].

### Possible Gender Specific Biological Mechanisms of Dioxin Effects

In animal experiments, exposure to 2,3,7,8-TetraCDD which has the highest TEF during pregnancy caused retardation of neurodevelopment in male offsprings [25], suggesting gender differences in the neurotoxicity of dioxins. We also found that perinatal 2,3,7,8-TetraCDD exposure induces decreased Ca^2+^/calmodulin-dependent protein kinase II α (CaMKIIα) activity in the limbic system of male rat offsprings which showed deficits in socio-emotional behavior, and increased CaMKIIα activity in female rat offsprings, which showed hyperactivity, but no socio-emotional deficits (unpublished data). Because CaMKII plays a role in regulating neurotransmission of γ-Aminobutyric acid (GABA) by phosphorylation of GABAergic receptors [26], [27], deficits in GABAergic neurotransmission might also contribute to alteration of behavior in 2,3,7,8-TetraCDD-exposed offsprings. Hays et al. [28] reported that maternal exposure to 2,3,7,8-TetraCDD gender-specifically altered the mRNA expression of glutamic acid decarboxylase (GAD) 67, which is an enzyme specific for the production of GABA, in the preoptic area of the neonatal rat brain, suggesting that 2,3,7,8-TetraCDD affects development of GABAergic neurons through the aryl hydrocarbon receptor (AhR) of which mRNA was co-localized with GAD67 mRNA in the brain region. These findings suggest that GABAergic neurons may be gender specific targets of 2,3,7,8-TetraCDD during development of sexually dimorphic brain regions in the hypothalamus and development of other brain regions including the limbic system.

### Dioxin Exposure and Decreased Body Weight Gain

In animal experiment, rodents treated using 2,3,7,8-TetraCDD at sublethal dose display a peculiar wasting syndrome characterized by decreased body weight gain, hypophagia, and feed refusal [29], [30]. Tuomisto et al. [31] reported that pretreatment with 2,3,7,8-TetraCDD completely blocked the effects of lesions in the ventromedial hypothalamus (VMH), which cause hyperphagia and obesity, suggesting an interaction between 2,3,7,8-TetraCDD and neuronal function in the VMH. Fetissov et al. [32] also reported that 2,3,7,8-TetraCDD exposure increased mRNA expression of some key hypothalamic neuropeptides involved in the regulation of body weight, with AhR immuno-reactivity in the nuclei of neurons. These findings suggest that 2,3,7,8-TetraCDD exposure affects neurological control of appetite and causes emaciation in exposed subjects. Recently, food intake has been reported to be regulated by a network in the brain that included not only VMH and the lateral hypothalamus, but also other sites in the limbic system of the brain. Involvement of GABAergic neurons in this network has been suggested in the control of feeding responses (both elicitation and suppression) by bidirectional interactions between sites [33]. Because much lower doses of 2,3,7,8-TetraCDD affect on development of GABAergic neurons [28], prenatal and postnatal exposure of dioxins at environmental exposure level might affect GABAergic neurons in the brain network that control food intake and induce appetite loss leading to decreased weight gain in human offspring. Future epidemiological studies are necessary to investigate the association between appetite and dioxin exposure in infants and children living in the present dioxin contaminated area.

### Dose–effect Relations and Long-term Effects

Body size and neurodevelopment scores in the <25, 25–50 and 50–75 percentile groups were similar. Significantly lower value for body size and neurodevelopment scores in the ≥75 percentile group were also found in boys compared with those in the other groups with <75 percentile of PCDDs/Fs-TEQ, suggesting that the threshold value of dioxin exposure for effects on infant growth may be approximately 75 percentile of PCDDs/Fs-TEQ (18.0 pg-TEQ/g fat), with no linear dose–effect relationship between infant growth and dioxin exposure. The upper range of background exposure levels in unsprayed areas was estimated to be 7.4 pg/g fat for PCDDs/Fs-TEQ based on data from a previous study [5]. Thus, the 75 percentile of PCDDs/Fs-TEQ found in the present subjects is much higher than the background level in Vietnam. However, the higher exposure group (defined by a cut off-value in the background exposure levels of 2,3,7,8-TetraCDD) displayed lower neurodevelopmental scores in the same population (unpublished data). Therefore, threshold values may differ among exposure markers. Further studies to estimate threshold values of dioxin effects on infant growth are required using benchmark dose analysis in a larger cohort of mother–infant pairs.

The duration of observation of the effects of dioxins on offspring growth is an interesting and important issue. In a Dutch cohort study [10], adverse effects of dioxin and PCB exposure on neurodevelopment were found in 7-month-old infants, but not in 18-month-old infants. However, among Yusho infants in Japan and Yucheng infants in Taiwan whose mothers accidentally consumed large amounts of PCBs and PCDFs through contaminated cooking oil, prenatal exposure to PCBs and dioxins resulted in adverse effects on body build and intellectual function until childhood or school age [34]–[36]. A recent study reported an association of dioxin and PCB exposure with reduced growth (BMI and height) among Russian boys aged 8–12 years [37]. The cohort examined here will be followed up until school age and the results reported in future studies.

### Limitations

The results of the present study must be interpreted within the context of its strengths and limitations. In the area exposed to dioxin from herbicides during the Vietnam War, this was the first birth cohort study carefully designed to clarify the association between perinatal dioxin exposure and health effects in a segment of the Vietnamese population. PCDDs/Fs-TEQ and not total TEQ including TEQ from dioxin like (dl)-PCBs, was used as an exposure marker because this study focused on dioxin contamination related herbicide exposure; thus, the level of dl-PCBs were not measured. However, contamination with PCBs at Da Nang airport has been reported [11], and elevated levels of PCBs in a variety of foods were documented in Vietnam [38], suggesting that TEQ estimation without dl-PCBs may be an under-estimation of toxicity in the present subjects. Therefore, dl-PCBs containing 4 non-ortho, 8 mono-ortho and 2 di-ortho PCB congeners were recently measured in four breast milk samples of mothers in the present study. The results indicated that dl-PCBs-TEQ was 12.8% (3.9 pgTEQ/g fat) of total TEQ, which is lower than that (38.1%; 2.1 pgTEQ/gfat) in 12 mothers in northern Vietnam and that (34.4%; 5.6 pgTEQ/g fat) in 134 Japanese mothers reported in another study [10]. These findings suggest that the contribution of dl-PCBs to total TEQ is not higher in this study area than those in other areas. Thus, PCDDs/Fs-TEQ can be used as an indicator of dioxin toxicity in this contaminated area.

Outcome effects included body size measurements (weight, height, and head and abdominal circumference), and neurodevelopment estimated using the Bayley III, which quantitatively and independently assesses various aspects of infant neurodevelopment. Because measurement of body size parameters other than weight is not common in Vietnam, detailed information on infant growth parameters, including BMI and head and abdominal circumference has been unavailable until this study. Because no standard of infant growth in Vietnamese children was available for comparison, the WHO standard was used to calculate z score. In addition, the results of the Bayley III testing in Vietnamese infants may be affected by differences in socioeconomic factors, customs, cultures, and languages because the Bayley III was developed and standardized for use in an American infant population. Therefore, developmental levels of individual Vietnamese infants could not be judged based on the scores of this test. However, in the present study, all testing was performed by the same examiner who was well trained in use of the Bayley III, and the examiner strictly followed the administration guidelines. Therefore, comparison among infants within the population should be reliable.

### Conclusions

Adverse effects of perinatal dioxin exposure on infant growth, including weight gain and neurodevelopment, were exhibited in Vietnamese residents of dioxin-contaminated areas where herbicides were stored during the Vietnam War. Health effects were gender-specific; boys were more susceptible. No linear dose-effect relationship was observed between PCDDs/Fs-TEQ levels and infant growth. A longer follow-up study of the present cohort in Vietnam is required to clarify health issues in the next generation related to dioxin exposure.
